# Effects of Non-Alcoholic Fatty Liver Disease on the Inpatient Outcomes of Patients Admitted for Atrial Fibrillation: An Analysis from the National Inpatient Sample Database (NIS 2016–2019)

**DOI:** 10.3390/jcdd13090436

**Published:** 2026-09-04

**Authors:** Xiuhong Lyu, Bolun Liu, Yiting Li

**Affiliations:** 1 Department of Hospital Medicine, Brown University Health, 593 Eddy St., Providence, RI 02903, USA; 2 Department of Hospital Internal Medicine, Mayo Clinic Health System, 1025 Marsh St., Mankato, MN 56001, USA; liu.bolun@mayo.edu; 3 Department of Gastroenterology, Gastro Health (Puget Sound Gastroenterology), 209 Lilly Rd. NE, Suite B, Olympia, WA 98506, USA; yitingli.med@gmail.com

**Keywords:** non-alcoholic fatty liver disease, NAFLD, atrial fibrillation, national inpatient sample, NIS, all-cause mortality, resource utilization

## Abstract

Background: Non-alcoholic fatty liver disease (NAFLD), which is recognized under the updated nomenclature as Metabolic Dysfunction-Associated Steatotic Liver Disease (MASLD), is increasingly prevalent in the United States and worldwide. Emerging evidence links NAFLD to an elevated risk of atrial fibrillation (AF), yet data regarding the impact of NAFLD on inpatient outcomes among patients admitted for AF remain limited. Methods: The National Inpatient Sample (NIS) from 2016 to 2019 was used to identify adult patients with a primary discharge diagnosis of AF (ICD-10: I48.x). Patients with concurrent NAFLD were compared to those without. Multivariable linear and logistic regression analyses were performed, adjusting for age, sex, race, insurance status, and Charlson Comorbidity Index. The primary outcome was inpatient all-cause mortality. Secondary outcomes included length of stay, inflation-adjusted hospital costs, discharge disposition, and in-hospital complications. Results: Of 1,891,479 weighted AF admissions, 13,840 carried a concurrent NAFLD diagnosis. Concurrent NAFLD was associated with longer length of stay (3.99 vs. 3.35 days, *p* < 0.001) (which is statistically significant though might be clinically insignificant) and higher hospital costs ($13,841.65 vs. $12,154.55, *p* = 0.046). No significant difference in inpatient mortality was observed. Conclusions: Concurrent NAFLD among AF hospitalizations is associated with greater resource utilization without a significant mortality difference, highlighting the importance of addressing this comorbidity to reduce the economic burden of AF-related hospitalizations.

## 1. Introduction

Non-alcoholic fatty liver disease (NAFLD), now recognized under the updated nomenclature as Metabolic Dysfunction-Associated Steatotic Liver Disease (MASLD) following the 2023 multi-society Delphi consensus and subsequent American Association for the Study of Liver Diseases (AASLD) practice guidance update [1], refers to the presence of hepatic steatosis with no other causes for secondary hepatic fat accumulation (e.g., heavy alcohol consumption) present [2]. It is a complex disease spectrum ranging from the more benign condition of non-alcoholic fatty liver (NAFL) to non-alcoholic steatohepatitis (NASH), which is at the more severe end of the spectrum [3]. The prevalence of NAFLD has increased worldwide, with a prevalence of 10–46% in the United States [4] and 25.2% worldwide [3,5]. Furthermore, NAFLD is frequently associated with or coexists with comorbidities such as obesity [6], insulin resistance, diabetes mellitus [5,7], hypertension, and hyperlipidemia. All of these are established risk factors for cardiovascular disease [8], including cardiac arrhythmias [9], such as atrial fibrillation.

The associations between NAFLD and atrial fibrillation have been highlighted recently, with most epidemiological studies demonstrating that NAFLD was associated with a higher risk of prevalent or incident atrial fibrillation [10,11]. One meta-analysis conducted by Wijarnpreecha et al. [12] showed that NAFLD patients had a two-fold increased risk of atrial fibrillation as compared to those without NAFLD. Mechanistically, it was thought that systemic inflammation might be an important mediator of plausible pathophysiological links between NAFLD and AF [13,14,15], though direct measurement of inflammatory biomarkers was not feasible in the current administrative database study. Pro-inflammatory cytokines like IL-1, IL-6, and TNF-α play a major role in the pathogenesis of NAFLD, which also plays a pivotal role in the pathogenesis of developing atrial fibrillation [16,17]. Furthermore, systemic inflammation has also been proposed as the pathophysiological link between obesity, metabolic syndrome, and NAFLD [6]. It has been well reported that atrial fibrillation patients with comorbid obesity, metabolic syndrome [18], and diabetes [19] would have worse clinical outcomes, including an increased risk of higher mortality [19].

While most studies focused on the risk (prevalence and incidence) of developing AF in NAFLD patients as presented above, data regarding the impact of NAFLD on the inpatient outcomes of patients admitted for atrial fibrillation are sparse. Given the shared common pathway involving the cardiovascular risk factors among obesity, metabolic syndrome, diabetes mellitus, NAFLD, and atrial fibrillation, we hypothesized that atrial fibrillation patients with concurrent NAFLD might have poorer inpatient outcomes as compared to patients without NAFLD. The current study retrospectively analyzed data from the National Inpatient Sample database from 2016 to 2019, aiming to elucidate the effect of NAFLD on the inpatient outcomes of patients admitted for atrial fibrillation, such as all-cause mortality, hospital length of stay, hospital total charges/costs, and discharge dispositions.

## 2. Materials and Methods

### 2.1. Data Collection and Patient Population

National Inpatient Sample (NIS) data from the online Healthcare Cost and Utilization Project Central Distributor (HCUP) were used for the study [Agency for Healthcare Research and Quality (AHRQ). National Inpatient Sample (NIS). Available at: https://hcup-us.ahrq.gov/nisoverview.jsp (accessed on 29 August 2026) [20]. The National Inpatient Sample (NIS) is the largest publicly available all-payer inpatient discharge database in the United States and consists of weighted data representing more than 35 million hospitalizations annually in the United States. All the datasets were purchased from the Online Healthcare Cost and Utilization Project Central Distributor (HCUP, Rockville, MD, USA). According to Brown University’s Human Research Protection Program self-assessment criteria, this study was determined to be exempt from Institutional Review Board review, as it involved only publicly available data containing no individually identifiable information. Informed consent was therefore not required.

In the current study, NIS discharge data from 1 January 2016 to 31 December 2019 were used. Discharges stored in this database represent a random sample of 20% of discharges from all the nonfederal hospitals in the United States [21], which were further stratified by hospital, ownership status, hospital location, census division, hospital teaching status, hospital bed size, etc. Patient-level and hospital-level trend weights were used to obtain national estimates.

The study population was generated retrospectively by querying the NIS for all hospital admissions of adult patients (ages equal to or more than 18 years old) from 1 January 2016 to 31 December 2019. Atrial fibrillation was identified using the International Classification of Diseases, Tenth Revision, Clinical Modification (ICD-10-CM) code I48.x as the primary discharge diagnosis, encompassing paroxysmal atrial fibrillation (I48.0), typical atrial flutter (I48.1x), other and unspecified atrial flutter (I48.2x), chronic atrial fibrillation (I48.1x), typical atrial flutter (I48.3), atypical atrial flutter (I48.4), and unspecified atrial fibrillation and atrial flutter (I48.9x). Non-alcoholic fatty liver disease (NAFLD) was identified as a secondary discharge diagnosis using ICD-10-CM code K76.0 (fatty change in liver, not elsewhere classified), which encompasses hepatic steatosis without other specified etiology.

It is important to note that the nomenclature of this disease entity has recently evolved. In 2023, a multi-society Delphi consensus proposed renaming NAFLD to Metabolic Dysfunction-Associated Steatotic Liver Disease (MASLD), subsequently adopted by the AASLD in updated practice guidance [1]. As this study utilized NIS data from 2016 to 2019, the legacy ICD-10 code K76.0 for NAFLD was applied throughout, and findings are interpreted in the context of this updated nomenclature.

### 2.2. Demographic Information, Clinical Variables, and Outcomes

Demographic information, including patient-level and hospital-level characteristics, such as age, sex, race, hospital region, hospital teaching status, hospital bed size, primary payer, and the household median income, was collected and examined across the atrial fibrillation cohort, then stratified by the presence or absence of NAFLD. Clinical variables, mainly comorbidities, were assessed using ICD-10-CM codes (please see Supplementary Table S1 for details of ICD-10 coding for each comorbidity). The variables collected were hypertension, diabetes, hyperlipidemia, vascular disease, which included coronary artery disease, ischemic heart disease, carotid artery disease, peripheral artery disease, atherosclerotic disease, chronic congestive heart failure, stroke/transient ischemic attack/systemic thromboembolism, obesity, metabolic syndrome, obstructive sleep apnea, chronic obstructive pulmonary disease (COPD), chronic kidney disease, liver disease other than NAFLD, anemia, hypothyroidism, tobacco use, alcohol use, drug use, and anticoagulation use. Charlson comorbidity scores [22,23] were also queried, which consisted of age, myocardial infarction, congestive heart failure, peripheral artery disease, cerebrovascular accident/transient ischemic attack, dementia, chronic obstructive pulmonary disease, connective tissue disease, peptic ulcer disease, liver disease, diabetes mellitus, hemiplegia, lymphoma, and AIDS. Primary outcomes included in-hospital all-cause mortality. Secondary outcomes included length of stay (LOS), overall inflation-adjusted total hospital charges and costs, and discharge disposition. Inpatient clinical course variables, including the ratio of undergoing an ablation procedure (using procedure code), undergoing pharmacological cardioversion, developing acute heart failure, acute myocardial infarction, acute kidney injury, undergoing hemodialysis, and progression to cardiogenic shock and cardiac arrest, were also evaluated.

### 2.3. Statistical Analysis

Continuous variables and categorical variables were summarized with descriptive statistics. Specifically, continuous variables were expressed with mean and standard error, while frequencies and percentages were used for categorical variables. Univariate analysis was undertaken for between-group unadjusted comparisons using the Chi-Square test for categorical variables and simple linear regression analyses for continuous variables. A weighted multivariable logistic or linear regression model was constructed to evaluate the effects of NAFLD on inpatient primary outcomes and secondary outcomes of Atrial fibrillation. The model was adjusted for age, sex, race, insurance status, and Charlson comorbidity score.

Total hospital charges were adjusted for inflation and normalized to US dollars in July 2022 using the Consumer Price Index inflation calculator published by the US Bureau of Labor Statistics. Furthermore, total hospital cost was generated from hospital total charges using the hospital-specific cost-to-charge ratio profile from HCUP. STATA 17 (StataCorp, College Station, TX, USA) was used for statistical analysis. *p* values < 0.05 were considered statistically significant.

## 3. Results

### 3.1. Basic Patient-Level and Hospital-Level Characteristics

From 1 January 2016 to 31 December 2019, we identified a total of 1,891,479 weighted hospitalizations with a primary discharge diagnosis of atrial fibrillation among patients aged more than 18 years, of which 13,840 (0.73%) had a secondary diagnosis or comorbid NAFLD. Demographic information was summarized in Table 1.

Among all the patients admitted for atrial fibrillation, the average age of patients was 70.57 years. About 49.19% of patients were female. A total of 81.67% of patients were white, 8.44% of patients were black, and 5.94% of patients were Hispanic. Others consisted of 3.95%. Patients with a primary diagnosis of AF and comorbid NAFLD were significantly younger (mean age 63.44 years vs. 70.62, *p* < 0.001) and comprised a lower ratio of the female sex (41% vs. 49.25%, *p* < 0.001), a lower proportion of white adults and black adults (86.39% vs. 90.14%, *p* < 0.001), but a higher proportion of Hispanic adults (9.33% vs. 5.91%, *p* < 0.001) when compared to those with a primary diagnosis of AF without NAFLD. A higher proportion of patients were admitted into hospitals in the West (20.05% vs. 15.23%, *p* < 0.001) in the AF-NAFLD group, but a lower proportion of patients were admitted into hospitals located in the Northwest region (18.5% vs. 19.78%), Midwest region (22.22% vs. 24.43%), and South region (39.23% vs. 40.69%) as compared to the AF-non-NAFLD group (*p* < 0.001). In total, 68.71% of patients were admitted into teaching hospitals in the AF-NAFLD group, which was significantly higher as compared to the AF-non-NAFLD group which was 65.82% (*p* = 0023). In the AF-NAFLD group, 48.43% of patients were from household incomes of the 51% quartile or higher, which was significantly higher than in the AF-non-NAFLD group, which was 45.56% (*p* = 0.0247). Regarding the insurance status, 51.32% of patients in the AF-NAFLD group were Medicare beneficiaries, which was significantly lower as compared to the AF-non-NAFLD group (51.32% vs. 68.82%, *p* < 0.001). The proportion of patients carrying Medicaid and private insurance, or self-pay, was significantly higher in the AF-NAFLD group than in the AF-non-NAFLD group (45.74% vs. 28.97%, *p* < 0.001). There was no significant difference regarding the admitting hospital bed sizes between the AF-NAFLD group and the AF-non-NAFLD group (*p* = 0.4375).

Regarding the comorbidity distribution, patients in the AF-NAFLD group had a higher prevalence of comorbid hypertension (81.32% vs. 79.17%, *p* = 0.0058), higher prevalence of comorbid dyslipidemia (54.48% vs. 51.63%, *p* = 0.0031), comorbid diabetes mellitus (37.54% vs. 28.8%, *p* < 0.0001), comorbid chronic heart failure (42.02% vs. 39.46%, *p* = 0.0068), comorbid obesity (43.39% vs. 21.63%, *p* < 0.0001), comorbid metabolic syndrome (1.12% vs. 0.22%, *p* < 0.001), comorbid sleep apnea (28.9% vs. 16.83%, *p* < 0.001), comorbid liver disease other than NAFLD (14.02% vs. 2.43%, *p* < 0.001), a higher proportion of tobacco use (43.61% vs. 38.5%, *p* < 0.001), alcohol use (20.38% vs. 5.19%, *p* < 0.001), and drug use (3.58% vs. 1.59%, *p* < 0.001), as compared to the AF-non-NAFLD group. Meanwhile, patients in the AF-NAFLD group had a lower prevalence of comorbid vascular disease (33.09% vs. 36.66%, *p* = 0.0001), chronic kidney disease (14.88% vs. 18.84%, *p* < 0.001), hypothyroidism (15.07% vs. 16.98%, *p* = 0.0077), and a lower proportion had anticoagulation (28.9% vs. 34.06%, *p* < 0.001) as compared to AF-non-NAFLD group. There were no significant differences in the prevalence of comorbid chronic obstructive pulmonary disease (*p* = 0.5611) and comorbid anemia (*p* = 0.0858) between the two groups.

### 3.2. Primary Outcomes (Including All-Cause Mortality and Resource Utilization (Length of Stay, Hospital Total Charges and Total Costs, and Discharge Dispositions)

Overall, the mean length of stay in the whole study population was 3.36 days. The mean inflation-adjusted total hospital charges were 52,547.41 dollars. The mean inflation-adjusted total hospital costs were 12,166.85 dollars. See Table 2 for details.

Before adjusting for covariates, patients in the AF-NAFLD group had a higher length of stay (3.99 vs. 3.35, *p* < 0.001), higher inflation-adjusted hospital charges (57,919.6 vs. 52,417.41, *p* < 0.001), and a higher inflation-adjusted hospital cost as well. After adjusting for age, sex, race, primary payer status, and Charlson comorbidity score, the length of stay of patients in the AF-NAFLD group was still significantly higher than that in the AF-non-NAFLD group (*p* < 0.001). The total hospital cost in the AF-NAFLD group was significantly higher than in the AF-non-NAFLD group after adjusting for covariates (*p* = 0.046). However, there was no statistically significant difference regarding the total hospital charges after adjusting (*p* = 0.575). Regarding all-cause mortality (see Table 2), there was no significant difference between the two groups before (0.72% vs. 0.85%, OR = 0.85, 95% CI 0.55–1.33) and after adjusting (aOR = 0.89, 95% CI 0.57–1.39, *p* = 0.608).

Regarding discharge disposition, overall, 74.55% of patients were discharged to home and self-care, 2.09% of patients were transferred to another acute care facility, 10.39% of patients were discharged to a skilled nursing facility, 11.85% of patients were discharged to home with healthcare, and about 1.12% of patients left against medical advice. Before adjustment, interestingly, there was a significantly higher ratio of patients discharged to home and self-care in the AF-NAFLD group as compared to the AF-non-NAFLD group (78.26% vs. 74.52%), a higher ratio of patients transferred to another acute care facility (2.37% vs. 2.09%), and a higher ratio of patients who left against medical advice (1.82% vs. 1.11%). However, after adjustment, there were no significant differences between the two groups regarding discharge disposition (*p* = 0.315).

### 3.3. Secondary Outcomes (Inpatient Clinical Course Outcomes)

Regarding the secondary outcomes (inpatient clinical course, see Table 3), patients in the AF-NAFLD group had a higher proportion of developing acute heart failure (21.32% vs. 18.64%, OR = 1.18, 95% CI 1.09–1.3, *p* = 0.0003), developing acute kidney injury (14.85% vs. 12.49%, OR = 1.22, 95% CI 1.1–1.36, *p* = 0.0002), or progressing into cardiogenic shock (1.19% vs. 0.6%, OR = 2, 95% CI 1.42–2.82, *p* = 0.0001), but a lower proportion undergoing ablation (5.17% vs. 6.55%, OR = 0.78, 95% 0.66–0.92, *p* = 0.0028), as compared to the AF-non-NAFLD group. There were no significant differences regarding the proportion of undergoing pharmacologic cardioversion (0.43% vs. 0.35%, OR = 1.23, 95% CI 0.7–2.14, *p* = 0.4695), developing acute myocardial infarction (2.49% vs. 3.07%, OR = 0.81, 95% CI 0.63–1.03, *p* = 0.0788), and proportion of undergoing hemodialysis (1.37% vs. 1.88%, OR = 0.73, 95% CI 0.52–1, *p* = 0.0558) and having a cardiac arrest (0.47% vs. 0.4%, OR = 1.17, 95% CI 0.68–2.03, *p* = 0.565). However, after adjusting for age, sex, race, primary payer status, and Charlson comorbidity score, there were no significant differences between these two groups regarding the rate of developing acute heart failure (aOR = 0.93, 95% CI 0.84–1.02, *p* = 0.107), acute kidney injury (aOR = 1.02, 95% CI 0.91–1.14, *p* = 0.714), and cardiogenic shock (aOR = 1.31, 95% CI 0.93–1.85, *p* = 0.126). There was still a significant difference regarding the rate of undergoing ablation between the two groups (aOR = 0.67, 95% CI 0.57–0.79, *p* < 0.001). Interestingly, the ratio of patients developing acute myocardial infarction (aOR = 0.61, 95% CI 0.48–0.77, *p* < 0.001) and undergoing hemodialysis (aOR = 0.38, 95% CI 0.27–0.53, *p* < 0.001) were significantly lower in the AF-NAFLD group as compared to the AF-non-NAFLD group.

## 4. Discussion

Our current study showed that patients admitted for atrial fibrillation with comorbid NAFLD had a longer length of stay and higher adjusted hospital costs, both before and after adjusting for covariates. However, there was no significant difference in all-cause mortality between these two groups before and after adjusting for covariates. Atrial fibrillation patients with comorbid NAFLD also had higher total hospital charges as compared to those without NAFLD, but the difference was not statistically significant after adjustment.

It was also shown here that atrial fibrillation patients with comorbid NAFLD were significantly younger than those without comorbid NAFLD (63.44 vs. 70.62, *p* < 0.001), which is in concordance with the previous finding by Gholitabar et al. [24], who showed that patients admitted for atrial fibrillation with comorbid NAFLD had a mean age of 62.7 as compared to those without NAFLD (mean age of 70 years old). In our study, AF-NAFLD patients were predominantly male and white adults, with a higher proportion of Hispanic patients as compared to the AF-NAFLD group. The AF-NAFLD group patients also had a higher ratio of Medicaid/private insurance/self-pay carriers, and a higher ratio of having a median household income above the 51st quantile, reflecting the association between AF-NAFLD and socio-economic factors. AF-NAFLD group patients had a higher ratio of comorbid hypertension, dyslipidemia, diabetes mellitus, obesity, metabolic syndrome, chronic heart failure, and sleep apnea, which was further supported by the finding that AF-NAFLD had a higher Charlson comorbidity score as compared to the AF-non-NAFLD group.

In line with the above findings, a growing body of evidence indicates that NAFLD is a multi-system disease [3,25,26]; its morbidity and mortality burden is not merely confined to the liver, but to other organ systems as well, affecting extra-hepatic organs and regulatory pathways [27]. For example, it was found that NAFLD increases the risk of diabetes mellitus [7], chronic kidney disease [27], and cardiovascular disease [8], including cardiac arrhythmias such as atrial fibrillation. A meta-analysis conducted by Gong et al. [9], elucidating the relationship between non-alcoholic fatty liver disease and cardiac arrhythmia, found that NAFLD was independently associated with a higher risk of atrial fibrillation (OR 1.71, 95% CI 1.14–2.57). Another prospective study (OPERA study) conducted by Karajamaki et al. [10] also found that NAFLD is independently associated with the risk of atrial fibrillation. Furthermore, there were studies indicating increased cardiovascular mortality in patients with comorbid NAFLD, although some studies also failed to find the associations [28]. Specifically, Minhas et al. conducted a national inpatient sample analysis on the effects of NAFLD on heart failure, finding that it was associated with increased mortality and longer length of stay as compared to those without NAFLD [29]. Reports regarding the effects of NAFLD on inpatient atrial fibrillation outcomes were sparse. In our current study, we found that atrial fibrillation patients with comorbid NAFLD had a longer length of stay in the hospital with higher total hospital costs before and after adjusting for covariates. These results were similar to Ali et al. [30], who conducted a retrospective study using national inpatient data in 2016 studying the effects of NAFLD on outcomes of patients admitted for Percutaneous Coronary Intervention, finding that those with NAFLD had a longer length of stay, were admitted at a younger age, and had significantly more cardiovascular comorbidities compared to those without NAFLD. We did not find any differences regarding all-cause mortality between these two groups. Similarly, the study conducted by Then et al. [28] using the National Sample Database concluded that NAFLD also did not worsen primary clinical outcomes (all-cause mortality) in patients who were admitted for acute myocardial infarction.

Our study also found that atrial fibrillation patients with comorbid NAFLD had higher total hospital costs as compared to those without NAFLD. Our finding was also in accordance with the report conducted by Allen et al. using large sample data from OptumLabs Data Warehouse [31], showing that the long-term cumulative healthcare cost of a NAFLD patient is 80% higher than that of a non-NAFLD control of similar age and metabolic comorbidities. Moreover, NAFLD patients experienced substantial increases in resource utilization, including imaging, hospitalizations, and laboratory tests, when compared to controls. They further concluded that given the increasing prevalence of NAFLD, the economic burden associated with it will considerably increase. Proactive management of this disease entity may help reduce resource utilization in the long run.

Our study showed that AF-NAFLD patients had significantly higher odds of developing acute heart failure, acute kidney injury, and cardiogenic shock during hospitalization compared to the AF-non-NAFLD group before adjustment for covariates. However, the differences became insignificant after adjusting for covariates. Interestingly, AF-NAFLD group patients had a lower odds ratio of undergoing ablation both before and after adjusting for covariates. Furthermore, it was found that AF-NAFLD group patients also had lower odds of developing acute myocardial infarction and undergoing hemodialysis after adjusting for covariates. These findings reflected the intricacy and complexity of the effects of NAFLD exerted during the in-hospital course of atrial fibrillation patients.

While there were reports focusing on elucidating the pathophysiologic link between cardiovascular disease and NAFLD [8], the underlying pathways associating these two disease entities (AF and NAFLD) remain to be determined. It was proposed that NAFLD and CVD are both manifestations of metabolic syndrome, with multiple complex pathways involved in the pathogenesis, including endothelial dysfunction, atherogenic dyslipidemia, systemic/vascular inflammation [8], altered glucose metabolism, coagulopathy, and altered gut microbiome. Furthermore, the interplay between NAFLD and atherosclerosis involves complex inflammatory and metabolic pathways, as recently highlighted by emerging evidence on the liver–heart axis and updated cardiovascular prevention perspectives [32,33]. Molecularly, inflammatory pathways mediated by IL-6, TNF-alpha, and CRP have been linked to NAFLD, with elevated IL-6 levels reported in NAFLD patients [34]. There were also reports linking inflammatory pathways involving IL-6 with atrial fibrillation [17]. Interestingly, in the study conducted by Lazzerini et al. [35], it was shown that systemic inflammation rapidly induces atrial electrical remodeling by down-regulating cardiac connexins. These changes might remarkably increase the risk of atrial fibrillation and related complications during the active inflammatory process. Moreover, the study conducted by Henningsen et al. [36] elucidated the prognostic impact of hs-CRP and IL-6 in patients with persistent atrial fibrillation treated with electrical cardioversion; it was found that low hs-CRP and IL-6 before cardioversion are associated with a lower risk of AF recurrence after cardioversion, indicating the role of the inflammatory process on atrial fibrillation outcomes. Similarly, the study conducted by Aulin et al. in the ARISTOTLE trial and RE-LY trial [37], both measuring the IL-6 levels in atrial fibrillation patients at different time points, demonstrated that IL-6 was associated with mortality, concluding that persistent systemic inflammatory activity, assessed by repeated IL-6 measurements, is associated with mortality independent of established clinical risk factors and other strong cardiovascular biomarkers in anticoagulated patients with AF.

Taken together with our current findings, we propose that, due to the shared underlying inflammatory pathway in these two disease entities as mentioned above, atrial fibrillation patients with concurrent NAFLD would have longer stays in the hospital, resulting in increased resource utilization and a perplexing in-hospital clinical course. The common pathways involved in the pathogenesis of NAFLD and AF might serve as a potential therapeutic target in the future.

## 5. Conclusions

Patients admitted for atrial fibrillation with comorbid NAFLD had a longer length of stay and higher adjusted hospital costs compared to those without NAFLD, without a significant difference in all-cause in-hospital mortality. These findings highlight NAFLD—now recognized under the updated nomenclature as MASLD—as a clinically meaningful comorbidity that increases the economic burden of AF-related hospitalizations independent of other confounders. Proactive screening for and management of NAFLD/MASLD in patients with atrial fibrillation may help optimize resource utilization and reduce healthcare costs.

## 6. Limitation

There are several limitations in the current study. Although national inpatient databases are increasingly used for clinical research, such studies are potentially susceptible to errors related to coding inaccuracies. The admissions were identified with a primary discharge diagnosis of atrial fibrillation, but it did not cover the patient population who were admitted into the hospital for other related diagnoses but were later found to have atrial fibrillation. Such as patients who were admitted to the hospital for syncope and were later found to have atrial fibrillation. In this scenario, such conditions (syncope) often ended up coding as the primary diagnosis. This might underestimate AF hospitalization frequency and its sequelae. On the other hand, this is a general and non-specific code for all atrial fibrillations; we were not able to identify more specifically whether it is new-onset atrial fibrillation or chronic persistent atrial fibrillation admitting for procedures such as Watchman device placement. Secondly, the current study was a retrospective observational study; it was impossible to establish causality from the current data. There was an association, but we could not say there was causation. Furthermore, the current study only focused on the inpatient outcomes of atrial fibrillation patients. A prospective cohort study will be needed to study the effect of NAFLD on long-term outcomes in atrial fibrillation patients, which will shed more light on the effects of atrial fibrillation patient outcomes.

It should be noted that the inflammatory status of the studied patients—including subclinical inflammation markers such as IL-6, TNF-alpha, and CRP—was not directly evaluated in this study due to the inherent limitations of the NIS administrative database. The observed differences in clinical outcomes between the AF-NAFLD and AF-non-NAFLD groups may, in part, reflect the underlying shared inflammatory pathways between these two disease entities, though this mechanistic link could not be directly confirmed from the current data.

## Figures and Tables

**Table 1:** Baseline characteristics of patients admitted to hospital for atrial fibrillation with and without NAFLD.

Variables	Total (N = 1,891,479)	A-Fib Without NAFLD (n = 1,877,639)	A-Fib with NAFLD (n = 13,840)	*p* Value
Geographic information				
**Age, years**	70.57	70.62 (70.56–70.68)	63.44 (62.97–63.91)	<0.001
**Female sex (%)**	49.19%	49.25%	41%	<0.001
**Race**				<0.001
White	81.67%	81.69% (81.29–82.08%)	79.38% (77.73–80.94%)	
Black	8.44%	8.45% (8.23–8.68%)	7.01% (6.1–8.03%)	
Hispanic	5.94%	5.91% (5.68–6.16%)	9.33% (8.21–10.59%)	
Others	3.95%	3.95% (3.78–4.12%)	4.28% (3.57–5.13%)	
**Region**				<0.001
Northwest	19.77%	19.78% (19.18–20.38%)	18.5% (16.96–20.14%)	
Midwest	24.28%	24.3% (23.7–24.91%)	22.22% (20.57–23.96%)	
South	40.68%	40.69% (39.99–41.39%)	39.23% (37.25–41.26%)	
West	15.27%	15.23% (14.79–15.69%)	20.05% (18.43–21.77%)	
**Teaching status**				0.0023
Teaching hospital	65.84%	65.82% (65.21–66.43%)	68.71% (66.79–70.58%)	
Non-teaching hospital	34.16%	34.18% (33.57–34.79%)	31.29% (29.42–33.21%)	
**Hospital bed size**				0.4375
Small	20.7%	20.71% (20.15–21.27%)	19.76% (18.16–21.47%)	
Medium	30.17%	30.16% (29.54–30.79%)	31% (29.15–32.91%)	
Large	49.13%	49.13% (48.42–49.85%)	49.24% (47.19–51.3%)	
**Primary payer**				<0.001
Medicare	68.7%	68.82% (68.58–69.07%)	51.32% (49.45–53.18%)	
Medicaid	6.21%	6.18% (6.05–6.31%)	10.71% (9.6–11.94%)	
Private/self-pay	22.88%	22.79% (22.56–23.02%)	35.03% (33.27–36.85%)	
Others	2.21%	2.21% (2.12–2.29%)	2.93% (2.37–3.63%)	
**Median household Income**				0.0247
1st–25th	27.33%	27.34% (26.83–27.85%)	26.28% (24.56–28.08%)	
26th–50th	27.09%	27.11% (26.7–27.52%)	25.29% (23.62–27.03%)	
51st–75th	24.92%	24.91% (24.53–25.3%)	25.98% (24.27–27.77%)	
76th–100th	20.66%	20.65% (20.09–21.21%)	22.45% (20.78–24.22%)	
**Past Medical History**				
Hypertension	79.19%	79.17% (78.99–79.36%)	81.32% (79.82–82.74%)	0.0058
Dyslipidemia	51.65%	51.63% (51.36–51.89%)	54.48% (52.59–56.36%)	0.0031
Diabetes	28.87%	28.8% (28.62–28.98%)	37.54% (35.75–39.36%)	<0.001
Vascular disease	35.63%	36.66% (36.44–36.87%)	33.09% (31.36–34.87%)	0.0001
Chronic heart failure	39.48%	39.46% (39.22–39.69%)	42.02% (40.17–43.89%)	0.0068
Stroke, TIA or systemic thromboembolism	10.5%	10.52% (10.4–10.63%)	7.84% (6.88–8.92%)	<0.001
Obesity	21.79%	21.63% (21.42–21.84%)	43.39% (41.51–45.29%)	<0.001
Metabolic syndrome	0.23%	0.22% (0.21–0.24%)	1.12% (0.79–1.59%)	<0.001
Sleep apnea	16.92%	16.83% (16.63–17.03%)	28.9% (27.19–30.67%)	<0.001
COPD	15.5% (15.35–15.64%)	15.49% (15.35–15.64%)	15.9% (14.57–17.31%)	0.5611
CKD	18.81% (18.65–18.98%)	18.84% (18.68–19.01%)	14.88% (13.58–16.29%)	<0.001
Liver disease other than NAFLD	2.51% (2.46–2.57%)	2.43% (2.38–2.48%)	14.02% (12.74–15.4%)	<0.001
Anemia	1.1% (1.06–1.13%)	1.1% (1.06–1.14%)	0.76% (0.5–1.16%)	0.0858
Hypothyroidism	16.97% (16.83–17.11%)	16.98% (16.85–17.13%)	15.07% (13.77–16.46%)	0.0077
Tobacco use	38.54% (38.26–38.81%)	38.5% (38.22–38.78%)	43.61% (41.77–45.46%)	<0.001
Alcohol use	5.3% (5.21–5.39%)	5.19% (5.1–5.27%)	20.38% (18.89–21.95%)	<0.001
Drug use	1.6% (1.56–1.65%)	1.59% (1.54–1.63%)	3.58% (2.93–4.36%)	<0.001
On anticoagulation	34.02% (33.69–34.36%)	34.06% (33.73–34.4%)	28.9% (27.23–30.64%)	<0.001
Charlson comorbidity score				<0.001
0–1	48.47% (48.25–48.68%)	48.68% (48.46–48.89%)	19.73% (18.27–21.27%)	
2–3	32.07% (31.91–32.23%)	31.93% (31.78–32.09%)	50.07% (48.17–51.97%)	
4–5	13.17% (13.05–13.3%)	13.13% (13–13.26%)	18.79% (17.37–20.29%)	
≥6	6.29% (6.2–6.39%)	6.26% (6.17–6.35%)	11.42% (10.26–12.68%)	

**Table 2:** Mortality and resource utilization comparison between the AF-NAFLD group and the AF-non-NAFLD group.

Variables	Total	AF-Non-NAFLD	AF-NAFLD	*p* Value
LOS, days	3.36 (3.34–3.37)	3.35 (3.34–3.37)	3.99 (3.85–4.14)	<0.001
Discharge disposition				0.315
Home, self-care	74.55% (74.33–74.78%)	74.52% (74.3–74.75%)	78.26% (76.71–79.73%)	
Transfer	2.09% (2.03–2.15%)	2.09% (2.03–2.15%)	2.37% (1.86–3.01%)	
SNF	10.39% (10.27–10.51%)	10.41% (10.28–10.53%)	8.12% (7.15–9.21%)	
Home healthcare	11.85% (11.69–12.01%)	11.87% (11.71–12.03%)	9.43% (8.4–10.58%)	
Against medical advice	1.12% (1.08–1.16%)	1.11% (1.08–1.15%)	1.82% (1.38–2.39%)	
Inflation-adjusted hospital charges, US $	52,547.41 ± 398.25	52,417.29 ± 398.36	57,919.6 ± 1447.29	0.575
Inflation-adjusted hospital costs, US$	12,166.85 ± 63.27	12,154.55 ± 63.02	13,841.65 ± 311.71	0.046

**Table 3:** In-hospital clinical outcomes, including all-cause mortality, of patients admitted for atrial fibrillation with and without NAFLD (unadjusted and adjusted).

Variables	Total	AF-Non-NAFLD	AF-NAFLD	Unadjusted OR	Unadjusted OR 95% CI	Adjusted OR	Adjusted OR 95% CI	*p* Value
Undergoing ablation	6.54%	6.55%	5.17%	0.78	0.66–0.92	0.67	0.57–0.79	<0.001
Pharmacological cardio-conversion	0.35%	0.35%	0.43%	1.23	0.7–2.14	1.12	0.64–1.99	0.687
Acute heart failure	18.66%	18.64%	21.32%	1.18	1.09–1.3	0.93	0.84–1.02	0.107
Acute myocardial infarction	3.07%	3.07%	2.49%	0.81	0.63–1.03	0.61	0.48–0.77	<0.001
Acute kidney injury	12.51%	12.49%	14.85%	1.22	1.1–1.36	1.02	0.91–1.14	0.714
Undergoing hemodialysis	1.87%	1.88%	1.37%	0.73	0.52–1	0.38	0.27–0.53	<0.001
Cardiogenic shock	0.6%	0.6%	1.19%	2	1.42–2.82	1.31	0.93–1.85	0.126
Cardiac arrest	0.4%	0.4%	0.47%	1.17	0.68–2.03	1.03	0.59–1.78	0.924
All-cause mortality	0.85%	0.85%	0.72%	0.85	0.55–1.33	0.89	0.57–1.39	0.608

## Data Availability

All data is available on the HCUP website: https://hcup-us.ahrq.gov/nisoverview.jsp (accessed on 29 August 2026).

## Supplementary Materials

The following supporting information can be downloaded at https://www.mdpi.com/article/10.3390/jcdd13090436/s1, Supplementary Table S1: ICD-10 codes for the classification of atrial fibrillation, non-alcoholic fatty liver disease, comorbidities, and outcome variables.
