# Supplementary material for: Effects of Non-Alcoholic Fatty Liver Disease on the Inpatient Outcomes of Patients Admitted for Atrial Fibrillation: An Analysis from the National Inpatient Sample Database (NIS 2016–2019)
Source: J Cardiovasc Dev Dis. 2026 Sep 4;13(9):436. doi: 10.3390/jcdd13090436 (PMC13607990; doi:10.3390/jcdd13090436)
Supplement: Supplementary file 1 [file jcdd-13-00436-s001.zip › jcdd-4434983-supplementary.pdf]

Table S1. ICD-10 codes for the classification of atrial fibrillation, non-alcoholic fatty liver disease, comorbidities, and outcome variables.

Diagnosis	ICD-10
Atrial fibrillation	I48.x
Non-alcoholic fatty liver disease	K76.0
HTN	I67.4, I11.x, I12.x, I15.x
hyperlipidemia	E78.x
Diabetes mellitus	E10.x, E11.x, E13.x
Vascular disease（coronary artery disease/ischemic heart disease, carotid artery disease, periphery artery disease or atherosclerotic disease）	I25.1, I25.2, I25.5, I25.6, I25.7, I25.8, I25.9, I25.10, I25.11, I25.82, Z95.1, Z95.8, Z95.9, I73.1, I73.9, I77.1, I79.0, K55.1, K55.8, K55.9, I70.x, I71.x, I20.x.
Chronic congestive heart failure	I11.0, I13.0, I13.2, 109.81, I25.5, I42.0, I42.5, I42.6, I42.7, I42.8, I42.9, I43, I50.x[a]
Stroke, systemic thromboembolism, transient ischemic attack (TIA)	I69.3, I69.9, I97.8, H34.0, H34.1, H34.2, H34.9, Z86.73, I63.x, I74.x, G45.x
Obesity	E66.0, E66.8, E66.9, E66.1, E66.01, E66.2, Z68.30, Z68.31, Z68.32, Z68.33, Z68.34, Z68.35, Z68.36, Z68.37, Z68.38, Z68.39, Z68.40, Z68.41, Z68.42, Z68.43, Z68.44, Z68.45.
Metabolic syndrome	E88.81.
Obstructive sleep apnea	G47.30, G47.31, G47.3, G47.39, G47.33, G47.36, G47.37.
Chronic obstructive pulmonary disease	J41, J41.0, J41.1, J41.8, J42, J43.0, J43.1, J43.2, J43.8, J43.9, J44, J44.9
Chronic kidney disease	N18, N18.3, N18.9, N18.1, N18.2, N18.4, N18.5, N18.6.
Liver disease other than NAFLD	K75.0, K75.1, K75.2, K75.3, K75.4, K75.8, K75.9, K77, K76, K76.1, K76.2, K76.3, K76.4, K76.5, K76.6, K76.7, K76.8, K76.9, K76.81, K76.82, K76.89, K70.x, k71.x, k72.x, k73.x, K74.x.
anemia	D50, D51, D52, D53, D55, D56, D57, D58, D59, D60, D61, D62, D63, D64.
hypothyroidism	E03.8, E03.9, E03.3, E03, E03.1, E03.0, E03.2, E89.
Tobacco use	Z87.891, F17.x
Alcohol use	Z87.898, G31.2, Z71.4, R78, G72.1, G62.1, F10.x
Drug use	Z27.15, F18, F19, F13, F14, F15, F16, F11, F12.x
On anticoagulation	Z79.01
Undergoing ablation	02580ZZ, 02583ZZ, 4A023FZ, 4A0234Z, 02K83ZZ
Undergoing pharmacological cardio conversion	3E030RZ, 3E033RZ, 3E040RZ, 3E043RZ, 3E050RZ, 3E053RZ, 3E060RZ, 3E063RZ.
Acute heart failure	I50.811, I50.21, I50.31, I50.23, I50.33, I50.813, I50.43, I50.41.
Acute myocardial infarction	I21.x, I22.x.

Acute kidney injury	N17.0, N17.1, N17.2, N17.8, N17.9.
Undergoing hemodialysis	5A1D60Z, 5A1D70Z, 5A1D80Z, 5A1D90Z, 5A1D00Z
Cardiogenic shock	R57.0
cardiac arrest	I46, I46.8, I46.9, I46.2, I97.12
